# An Analysis of Diffracted Mode Outcoupling in the Context of Optical Gain Measurements of Organic Thin Films: A Diffracted Emission Profile Method

**DOI:** 10.3390/mi17020153

**Published:** 2026-01-23

**Authors:** Thilo Pudleiner, Jan Hoinkis, Christian Karnutsch

**Affiliations:** 1 Research Group Integrated Optofluidics and Nanophotonics (IONAS), University of Applied Sciences Karlsruhe, 76133 Karlsruhe, Germany; thilo.pudleiner@h-ka.de; 2 Faculty of Electrical Engineering and Information Technology, University of Applied Sciences Karlsruhe, 76133 Karlsruhe, Germany; jan.hoinkis@h-ka.de

**Keywords:** organic DFB laser, amplified spontaneous emission (ASE), optical gain analysis, (co-)polymers, F8BT, diffraction grating

## Abstract

The sustained interest in efficient, low-cost, and straightforward-to-manufacture lasers has prompted intense research into organic semiconductor laser emitter materials in recent decades. The main focus of this research is determining the optical gains and losses of amplified spontaneous emission (ASE) in order to describe materials by their amplification signature. A method that has been used for decades as the standard technique for determining gain characteristics is the variable-stripe-length (VSL) method. The success of the VSL method has led to the development of further measurement techniques. These techniques provide a detailed insight into the nature of optical amplification. One such method is the scattered emission profile (SEP) method. In this study, we present an extension of the SEP method, the Diffracted Emission Profile (DEP) method. The DEP method is based on the detection of ASE by partial decoupling of waveguide modes diffracted by a one-dimensional grating integrated into a planar waveguide. Diffraction causes a proportion of the intensity to exit the waveguide, transferring the growth and decay process of the waveguide mode to the transverse mode profile of the diffracted mode. In the present article, an approach to determine the amplification signature of an organic copolymer is presented, utilizing partial decoupled radiation.

## 1. Introduction

Lasers have become an indispensable part of modern optical sensor technology and spectroscopy. The majority of lasers are nowadays based on inorganic semiconductors and doped crystals, which serve as the emitter materials. In contrast, organic semiconductors offer several advantages over inorganic materials, including ease of processing, mechanical flexibility, reduced toxicity, and excellent biocompatibility [1]. π-conjugated molecules or polymers are organic semiconductors that emit light over an extensive range of wavelengths, displaying high photoluminescence quantum efficiency and a large stimulated emission cross-section. Organic semiconductor-based lasers are notable for their broad tunability [2], miniaturizability [3,4,5], convenience, and cost-effectiveness in manufacturing. Consequently, these characteristics render them a subject of paramount interest in the realm of miniaturized analytical systems and microfluidic platform devices.

The utilization of distributed feedback (DFB) resonator structures has become a well-established convention for these systems. The deposition of organic semiconductors as thin films on periodic substrates is a straightforward process. Radiation propagating in waveguide mode is scattered by periodic corrugation and can combine coherently to create a “Bragg-scattered” wave [6]. Higher-order resonators can provide output coupling in addition to feedback. Such resonator designs enable single-mode operation while offering a high degree of integration into sensing systems [3,7,8].

The condition for lasing can be expressed most conveniently by requiring that the optical gain surpasses all losses, such as those arising from absorption, scattering, or outcoupling. In this context, the analysis of laser emitter materials primarily focuses on the amplification phenomenon, as the achievable optical gain is a key factor determining the efficiency of a laser. A widely used technique for quantifying optical amplification is the investigation of amplified spontaneous emission (ASE). ASE refers to the process in which spontaneously emitted photons undergo amplification by stimulated emission while propagating through the emitter material. The characteristic ASE behavior provides access to key gain parameters, such as the stimulated emission cross-section and the material’s amplification properties.

An early method to determine optical amplification in laser emitter materials is the variable-stripe-length (VSL) method. The VSL method was first introduced by Shaklee in 1973 [9] as a direct measurement technique for optical gain in thin-film semiconductors. While originally developed for inorganic semiconductors, it has since been applied to a wide range of material classes, including organic compounds [10,11,12], colloidal nanocrystals [13], and hybrid organic–inorganic halide perovskites [14]. In the VSL method, a thin planar layer of the emitter material is homogeneously excited in the shape of a stripe. The emission is collected at the edge of the sample while the excitation stripe length is systematically varied. Due to the homogeneous and stripe-shaped excitation, the system can be approximated as a one-dimensional linear amplifier. In this description, the growth of the emission intensity $I_{\left(\lambda \right)}$ along the stripe is governed by Equation (1) [12].

(1) $$\frac{\partial I\left(\lambda \right)}{\partial z}=g\left(\lambda \right)\cdot I\left(z,\lambda \right)-\alpha \left(\lambda \right)\cdot I\left(z,\lambda \right)+g\left(\lambda \right)\cdot \eta \left(\lambda \right)$$

where *z* is the position along the stripe, *g* denotes the optical gain, α represents the optical losses, and the parameter η is proportional to the spontaneous emission. In practice, the VSL method relies on recording the emission intensity as a function of the excitation stripe length and extracting the net gain (g−α) from the exponential growth of the signal. The corresponding differential equation describing the intense growth (see Equation (1)) can be solved by integration over the pump stripe length. Applying the boundary condition I(z=0)=0 yields an analytical expression for the intensity evolution, as shown in Equation (2).

(2) $$I\left(\lambda ,z\right)=\frac{g\left(\lambda \right)\cdot \eta \left(\lambda \right)}{g\left(\lambda \right)-\alpha }\cdot \left(e^{\left(g\left(\lambda \right)-\alpha \right)\cdot z}-1\right)$$

This common method has the advantage that a relative intensity measurement is sufficient to estimate the net gain. This analytical approach is based on the assumption of a superlinear relation; i.e., the gain exceeds the total optical losses (g−α>0) [15]. In cases where the optical losses dominate (g−α<0), no exponential increase in intensity is expected, and the emission remains in a sublinear regime. When considering a small segment in the location, the intensity generated by spontaneous emission and subsequently amplified is entirely compensated (or dissipated) by the losses so that no net amplification remains. The differential equation (Equation (1)) reduces to Equation (3) because the gain is insufficient to overcome the losses.

(3) $$\frac{\partial I\left(\lambda \right)}{\partial z}=\eta \left(\lambda \right)\cdot g\left(\lambda \right)$$

This equation can likewise be solved by integration over the pump stripe length. Applying the boundary condition I(z=0)=0 yields a linear expression for the intensity evolution, as shown in Equation (4).

(4) $$I\left(\lambda ,z\right)=\eta \left(\lambda \right)\cdot g\left(\lambda \right)\cdot z$$

This transition from superlinear behavior to sublinear behavior is used in some studies to estimate optical attenuation by fitting an effective negative gain in the sublinear regime [16]. More frequently, the optical losses are determined in a separate measurement, in which the edge emission is recorded as a function of the distance between the excited stripe and the sample edge [10,11,17,18]. For the commonly used exponential-growth approach, no meaningful gain value can be extracted in the sublinear regime. In some reports, this regime (g<α) constitutes a substantial portion of the measured range, exceeding two-thirds of the data interval considered (see, e.g., [11,18,19]). In such cases, the applicability of exponential fits becomes inherently limited, as the method relies on the presence of a positive net gain to yield physically interpretable results. For this reason, we present here a measurement approach that enables an estimation of the gain even within the sublinear regime. This approach is conceptually related to the scattered emission profile (SEP) method [20]. The experimental configuration, including the sample geometry and optical excitation scheme, is identical to that of the VSL method. To gain spatially resolved information on the radiation intensity along the pump stripe, scattered light originating from inhomogeneities at the planar waveguide interface is collected using an imaging spectrometer. The measurement setup thus closely resembles that of a waveguide attenuation experiment. As demonstrated in our previous work [21], this approach can be extended by integrating an optical diffraction grating into the sample geometry, enabling the detection of diffracted waveguide modes instead of scattered emission. In contrast to established methods such as the VSL and SEP techniques, an estimation of the net optical gain cannot be derived in the superlinear regime of the emitted intensity along the excitation stripe in the case of the DEP method. This is due to the comparatively high outcoupling losses introduced by the integrated diffraction grating.

According to Equation (2), the net gain appears in the exponent of the intensity expression. This formulation has the advantage that an estimation of the gain can be performed using relative intensity values, without requiring absolute signal calibration. In contrast, a gain estimation based on a linear intensity increase requires both an evaluation of the spontaneous emission contribution $\eta_{\left(\lambda \right)}$ and an accurate determination of the absolute emitted intensity. In contrast to VSL and SEP approaches, the DEP method uniquely exploits the spectral dependence of optical outcoupling, i.e., the extraction of individual waveguided modes into well-defined directions. This characteristic enables a direct separation of the emitted intensity into diffracted waveguided modes and spontaneously emitted contributions, thereby allowing optical amplification to be assessed even in loss-dominated regimes, which are not accessible using SEP or VSL methods. In the present study, this approach is further developed and applied to assess optical amplification in organic thin-film emitters, providing a complementary perspective to established measurement techniques.

Beyond their formal differences, the VSL, SEP, and DEP methods differ in experimental access and applicability. Conventional VSL measurements rely on edge-emitted radiation and therefore require well-defined and accessible sample edges, whereas both SEP and DEP probe the emission through the sample surface. The SEP method allows gain extraction from relative intensity measurements as long as optical amplification exceeds losses. However, in regimes corresponding to a negative net gain, SEP no longer provides unambiguous gain information. In contrast, the DEP method exploits the discrete angular and spectral outcoupling of individual waveguided modes, enabling gain-related information to be extracted from relative intensities even when the net gain is negative. This mode-resolved detection method constitutes a decisive advantage of DEP, although at the expense of increased complexity of the sample configuration.

## 2. Experimental Setup

In order to investigate the amplification behavior of organic thin-film emitters in a diffractive waveguide configuration, a sample geometry was fabricated and characterized. The experimental procedure can be divided into four parts: the description of the sample geometry and its fabrication, the optical excitation scheme, the propagation and diffraction of waveguide modes within the structure, and finally the detection of the emitted radiation. This section first introduces the sample design and fabrication process before addressing the optical setup and the measurement methodology.

**Figure 1:**
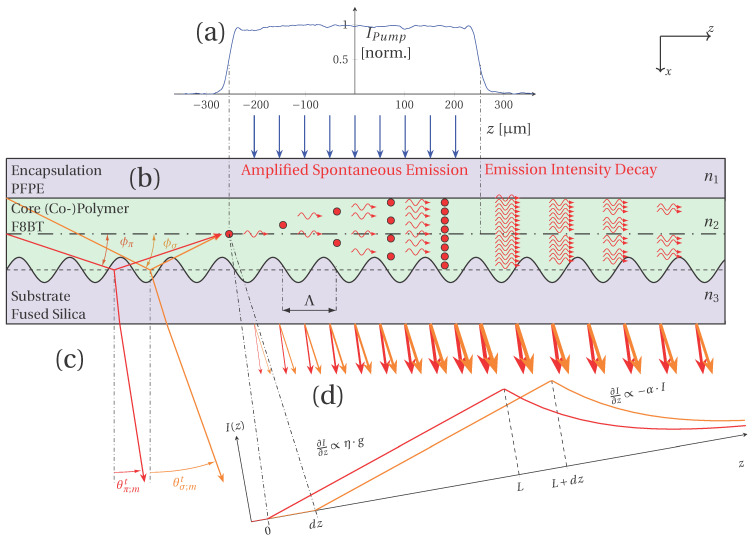
Sample setup and emission propagation: (**a**) UV pump radiation through the cladding layer. The diagram illustrates the normalized pump intensity as a function of the z position. Intensity is measured with a CMOS sensor placed at the sample position. (**b**) A cross-sectional view of the organic thin-film sample (not to scale). It consists of a sandwich structure comprising an encapsulation layer (PFPE), a core layer (F8BT) and a substrate (fused silica). A one-dimensional relief grating with the periodicity Λ is etched into the surface of the substrate. Mode propagation in the core of the waveguide is indicated from left to right. (**c**) Plane-wave propagation is outlined by the wave vectors for TE (orange) and TM (red). The wave vectors of a diffracted wave on the line grating are illustrated for the first transmitted diffraction order for each polarization direction (TE (orange) and TM (red)). (**d**) The emission of the sample by partially decoupled waveguide modes is illustrated by red and orange arrows. The diagram illustrates the expected increase in intensity for each polarization direction resulting from a homogeneous pumped sample and the anticipated decrease due to linear decay.

The schematic sample setup is depicted in Figure 1. The structure consists of three functional layers: the substrate with a surface relief grating, the active polymer emitter layer, and a protective encapsulation layer. The substrate is made of fused silica and incorporates a one-dimensional corrugation with a periodicity of Λ= 280 nm and a modulation depth of approximately 20 nm. As the active layer, the conjugated (co-)polymer poly[(9,9-dioctylfluorenyl-2,7-diyl)-co-(1,4-benzo-2,1’,3-thiadiazole)] with 10% benzothiadiazole units (commonly referred to as F8BT) was deposited by spin coating from a toluene solution, resulting in a film thickness of t= 180 nm ± 19 nm, as determined by laser confocal microscopy on a reference scratch. To prevent photooxidation during optical excitation, the film was encapsulated with a PFPE (perfluoropolyether) overlayer. The complete optical transparency of the multilayer design offers two practical advantages: it allows for optical pumping of the active layer through encapsulation using a UV source, and it enables efficient extraction of the emitted radiation through the transparent, fused silica substrate. The sample geometry resembles a misaligned DFB resonator, in which the grating periodicity is too small to provide resonance for the second-order DFB condition while being too large to enable first-order resonance under ASE conditions [21,22]. Within this configuration, the waveguide geometry allows guidance of first-order modes generated by spontaneous and stimulated emissions. These propagating modes are progressively coupled out at the diffraction grating into a diffracted mode.

(5) $$n_{2/3}\cdot \sin\left(\theta_{\sigma /\pi ,m}^{ref/trans}\right)=n_{2}\cdot cos\left(\phi_{\sigma ,\pi }\right)+\frac{m\cdot \lambda_{0}}{\Lambda }$$

The decoupling direction of the diffracted mode can be described by the diffraction equation (Equation (5)). In this expression, $n_{2}$ denotes the refractive index of the copolymer, $n_{3}$ is the refractive index of the corrugated substrate layer, $\lambda_{0}$ represents the free-space emission wavelength of the mode, Λ is the grating periodicity, *m* is the diffraction order, $\phi_{\sigma ,\pi }$ is the wave propagation angle, and $\theta_{\sigma /\pi ,m}^{ref/trans}$ is the diffraction angle of the reflected or transmitted order. The prediction of the diffraction angles can be obtained from the discrete eigenvalues of the propagation constant of the guided modes in the planar waveguide for a given polarization state [21]. Here, σ-polarization corresponds to the transverse electric (TE) mode, where the electric field is oriented perpendicular to the plane of incidence, whereas π-polarization corresponds to the transverse magnetic (TM) mode, with the magnetic field perpendicular to the plane of incidence. In addition to the geometrical and material parameters—such as the refractive indices of the layers and the core thickness—the propagation characteristics depend solely on the wavelength and the polarization of the guided mode. The relationship between wavelength and diffraction angle is nearly linear for the present waveguide geometry, resulting in a wavelength-dependent emission direction in the ASE regime.

To quantitatively predict the diffraction behavior, we calculated the discrete eigenvalues of the planar waveguide using the refractive indices of the individual layers at λ = 550 nm and $n_{1}$ = 1.28 [23] for the encapsulation, $n_{2}$ = 2.54 for the active copolymer film, and $n_{3}$ = 1.46 [24] for the corrugated substrate. The calculation was iteratively refined using the experimentally observed diffraction directions of the emitted modes, ensuring consistency between the modeled propagation constants and the measured decoupling angles. Within the emission range of the active material (480–650 nm), the resulting eigenvalue analysis yields solutions for the fundamental guided orders of both polarization states, specifically the TE0 and TM0 modes.

The partial outcoupling of a propagating mode within the waveguide is schematically illustrated in Figure 1. The emission behavior can be separated into two spatial regions: in the pumped region, the guided mode experiences material-specific amplification, whereas in the unpumped region, the mode decays due to the absence of a gain and spontaneous emission. This spatial separation of growth and decay constitutes the basis for our subsequent measurement approach, in which the emission is analyzed to extract information on the propagation and amplification characteristics of the waveguide modes.

**Figure 2:**
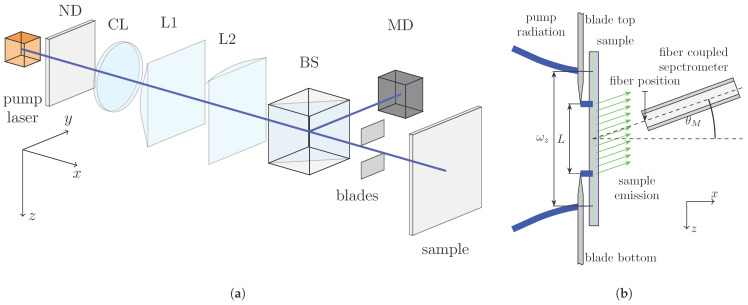
Schematic representation of the pump and measurement setup: (**a**) The sample is excited using a UV pump laser. The elliptical TEM_00_ profile of the pump beam is converted into a top-hat-like profile by razor blades. ND: neutral density filter, CL: collimator lens, L1: cylinder lens (*z* dimension), L2: cylinder lens (*y* dimension), BS: beam splitter, and MD: monitor diode. (**b**) Cross-sectional sketch of the sample and the sample emission measurement setup. The fiber of a fiber-coupled spectrometer is positioned close to the sample to detect the sample emission. The fiber is inclined at a measurement angle of $\theta_{M}$ relative to the sample normal. The fiber can be moved along the *z* dimension.

Figure 2a,b show the configuration of the pump and detection setup. The sample is excited by a pulsed frequency that is tripled Nd:YAG laser (FTSS355-Q2; CryLas, Berlin, Germany) with a pulse duration of 1.9 ns and operating at a wavelength of 355 nm. An elliptical pump spot is formed using a collimator lens in combination with two cylindrical lenses. At the sample position, the TEM_00_ pump profile exhibits an ellipse-like shape with a beam radius of $\omega_{z}=397.4\;\mu$m along the major axis and $\omega_{y}=31.6\;\mu$m along the minor axis. To achieve a homogeneous pump intensity along the *z*-direction, the beam is shaped by two razor blades that remove the off-axis intensity tails of the Gaussian profile, approximating a top-hat distribution. The blades are positioned in close proximity to the sample (d<0.5 mm) to minimize diffraction effects. The beam profile measured behind the blades yields a full-width-at-half-maximum (FWHM) length of L=505μm with a nearly uniform intensity distribution (as depicted in Figure 1a). Neutral density filters in the beam path are used to adjust the pump power. The incident power is monitored via a beam splitter and a calibrated photodiode, with an energy meter positioned at the sample location for calibration.

The emission from the sample is analyzed using the detection setup illustrated in Figure 2b. Outcoupled radiation is collected with a fiber-coupled spectrometer (USB2000; Ocean Optics, Ostfildern, Germany) connected to a multimode optical fiber with a core diameter of 25 μm. Owing to the numerical aperture of the fiber (NA = 0.1), the acceptance angle is limited, which restricts the transmitted spectral range of the diffracted emission to approximately 60 nm. As depicted in Figure 2a, the fiber is positioned at an inclination angle of $\theta_{M}$ with respect to the sample surface, corresponding to the diffraction angle of the observed emission order. To ensure efficient light collection and minimize spatial averaging, the distance between the sample and the fiber tip is kept as small as possible. The spectrometer is operated with an integration time of 1 s for all measurements. Given the pump laser repetition rate of 1 kHz, each recorded spectrum therefore corresponds to integration over 1000 excitation pulses.

## 3. Results

The experimental results demonstrate how the detection of the sample emission under systematic variation of the pump energy can be employed to characterize the amplification signature of the organic polymer. The evaluation procedure is divided into three parts: detection of the sample emission, processing of the measurement data, and numerical analysis.

The choice of sample design is essential for enabling a clear interpretation of the diffracted emission profiles. The polymer film is deposited on a substrate that contains a lateral array of gratings with different grating periodicities. Since all structural and optical parameters remain fixed for a given sample, the diffraction direction of the outcoupled waveguide mode depends solely on the grating periodicity. For the present investigation, a grating period of 280 nm was selected. This configuration ensures single-mode waveguiding, enables the appearance of a first-order diffraction channel within the ASE spectral range of F8BT, and avoids the occurrence of resonant features in the same spectral region.

### 3.1. Detection of the Sample Emission

**Figure 3:**
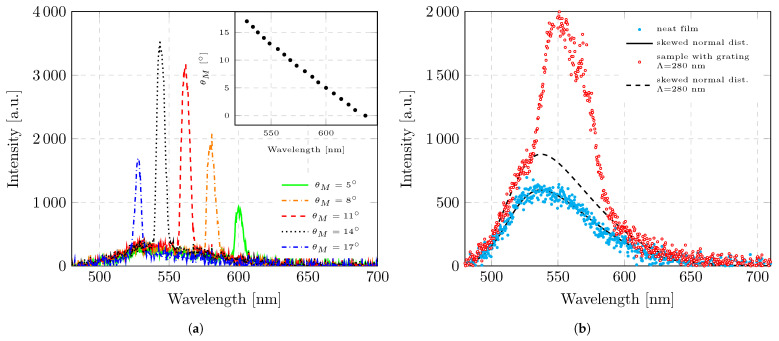
Analysis of sample emission and effects of the integrated grating when Λ = 280 nm. (**a**) Sample emission intensity as a function of emission wavelength for various measurement angles ($\theta_{M}$). The pump’s energy density remains constant for all measurements (E/A = 52.9 μJ/cm^2^). For spectral acquisition, a thick optical fiber (core diameter = 600 μm) equipped with a fiber collimator was positioned at a large distance from the sample ($d_{m}$ = 15 cm). The inset shows the measurement angle $\theta_{M}$ as a function of the peak emission wavelength corresponding to the maximum emission intensity. (**b**) Sample emission intensity as a function of the emission wavelength for a sample without an integrated grating and for a sample with an integrated grating when Λ = 280 nm. The measurement angle ($\theta_{M}$ = 11.8°) and the pump energy density (E/A = 68.6 μJ/cm^2^) are kept constant. A thin optical fiber (core diameter = 25 μm) without a collimator was used for the measurement. The fit of a skewed normal distribution is plotted for both sets of measurement data.

The modal emission of the sample exhibits pronounced angular dependence. Mode decoupling within the waveguide is governed by the layer thickness, refractive indices, grating periodicity, and diffraction angle, as described by Equation (5). Figure 3a shows the sample emission recorded at different measurement angles. The detection of the sample emission is performed at a large distance from the sample, representing the far-field regime. The emission consists of isotropic photoluminescence and a set of discrete diffracted modes originating from guided propagation within the waveguide. The isotropic photoluminescence shows no variation with respect to the measurement angle. In contrast, increasing the measurement angle $\theta_{M}$ results in a shift of the diffracted-mode emission toward shorter wavelengths. As shown in the inset, the measurement angle is plotted as a function of the peak wavelength of the diffracted modes, revealing a linear relationship. The use of a polarizer in the experimental setup revealed that the diffracted modes are linearly polarized, with the electric field oriented perpendicular to the plane of incidence. Consequently, the emission can be assigned to a TE mode. A TM mode could not be separated in the present measurement. However, if both the TE and TM modes were emitted by the sample, they could in principle be selectively detected by rotating the polarizer such that its transmission axis aligns with the respective polarization direction.

To analyze ASE, the measurement angle is selected based on the expected ASE emission range of F8BT. According to the literature, the ASE of the employed polymer, F8BT, is expected to occur around $\lambda_{\mathrm{ASE}}=560$ nm ± 15 nm [25,26]. Therefore, for all subsequent measurements, the measurement angle was fixed at $\theta_{M}=$11.8°. To investigate the near-field emission of the sample, the fiber collimator was removed, and an optical fiber with a core diameter of 25 μm was positioned at a small distance of approximately 0.5 mm from the sample surface. In this configuration, the number of detectable diffracted modes increases compared to that using far-field detection. Due to the reduced distance to the sample, all diffracted modes lying within the acceptance angle of the fiber are coupled into the waveguide of the fiber. As a result, the spectral bandwidth of the detected diffracted emission increases, as illustrated in Figure 3b. Furthermore, the reduced detector-to-sample distance leads to a stronger weighting of the isotropic emission component of the film, which contributes more prominently to the detected signal under near-field conditions.

The detection of the decoupled modes was performed by raster scanning an optical fiber along the excited region of the sample. The fiber, mounted on a motorized translation stage, was incrementally displaced along the *z*-axis in steps of 50 μm (see Figure 2b).

### 3.2. Processing of the Measurement Data

The detected radiation from the sample consists of a superposition of diffracted waveguide modes and the photoluminescence (PL) of the copolymer. PL represents an isotropic emission component that does not depend on the grating structure or the detection angle. Consequently, its spectral shape remains constant, while its absolute amplitude may vary as a scalar factor depending on the collection position along the excited region. In the angularly resolved measurements shown in Figure 3a, PL manifests as an identical, angle-independent background contribution. Figure 3b compares the emission spectra of a neat film and a sample incorporating a grating with Λ = 280 nm. The neat film’s spectrum is used to quantify the PL contribution in the total measured emission. For this purpose, PL is fitted using Azzalini’s skew-normal distribution [27].

(6) $$f\left(\lambda ;A,\xi ,\sigma ,\beta \right)=\frac{2\cdot A}{\sigma }\cdot \phi \left(\frac{\lambda -\xi }{\sigma }\right)\cdot \Phi \left(\beta \cdot \frac{\lambda -\xi }{\sigma }\right)$$

Here, λ denotes the emission wavelength, ξ is the mean, σ is the standard deviation, β is the skewness parameter, and *A* is an amplitude factor used for scaling. The functions ϕ and Φ represent the standard normal probability density function and its cumulative distribution function, respectively. The neat film’s spectrum was first fitted to determine the parameters ξ, σ, and β, which characterize the general PL emission. These parameters were found to remain constant across different positions along the pump stripe, confirming the spatial uniformity of the PL. The resulting fit function is shown in Figure 3b.

Using the parameters ξ, σ, and β obtained from the fit of the uniform PL (neat film), the emission spectrum of a sample incorporating a grating was subsequently analyzed. These three parameters were kept fixed to represent the invariant PL contribution, while only the amplitude factor *A* was adjusted to account for the overall intensity scaling at different measurement positions. To prevent any influence from the diffracted waveguide modes, the spectral region between 526 nm and 596 nm, where the guided modes appear, was excluded from the fitting procedure. The corresponding fit to the grating sample is also shown in Figure 3b. Fixing ξ, σ, and β ensures that the photoluminescence background is treated as a material-specific and spatially invariant contribution to the total emission. This approach allows for a clear separation between the isotropic PL component and the additional spectrally narrow features associated with diffracted and amplified modes. The validity of this method relies on the assumption that the copolymer layer is similar across the sample—i.e., prepared under the same conditions and exhibiting similar structure parameters—which is ensured as all measurements, including those of the neat film, were performed on the same sample substrate.

**Figure 4:**
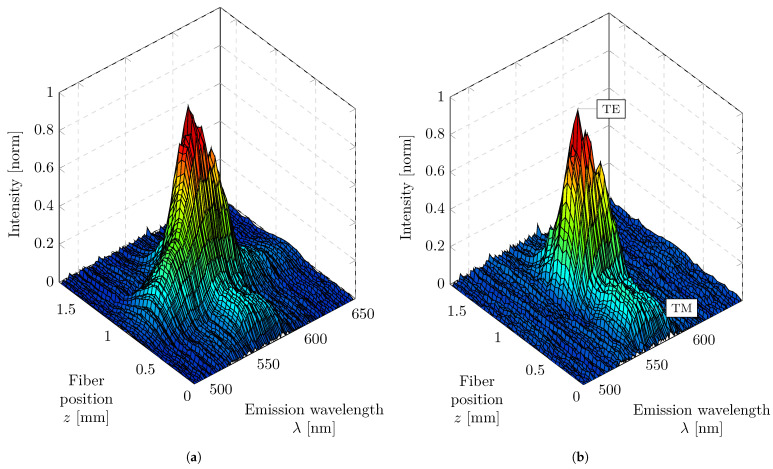
Spatial and spectral emission behavior of the sample and processing of the measurement data. The sample contains a grating periodicity of Λ = 280 nm and is excited during the measurement at a pump energy density of E/A = 68.6 μJ/cm^2^. (**a**) Sample emission measurement data as a function of fiber position and emission wavelength. (**b**) Emission measurement data as a function of fiber position and emission wavelength, numerically corrected for isotropically emitted spontaneous emission.

After determining the PL parameters, the fitted PL contribution was subtracted from the measured spectra to isolate the intensity associated with the diffracted modes. Figure 4a shows the raw spatially resolved emission data as a surface plot, illustrating the measured intensity as a function of fiber position and emission wavelength. The corresponding data after subtraction of the fitted PL background are presented in Figure 4b.

In the employed measurement geometry, the downward-tilted optical fiber detects the diffracted modes originating from waveguide modes that propagate from the lower edge to the upper edge of the sample. The emission intensity of these diffracted modes increases progressively with fiber position along the excitation stripe, corresponding to the amplification of the guided modes within the pumped region. Outside the pumped area, the modal intensity decreases exponentially, reflecting the attenuation of the waveguide modes in the absence of optical gain. This representation thus reveals the spatial and spectral evolution of the guided modes along the excitation stripe, with the PL background effectively removed. In addition to the primary modal progression, the data reveal a second, spatially shifted intensity evolution. This weaker progression is attributed to a TM mode that also fulfills the diffraction condition but exhibits a smaller diffraction angle than the dominant TE mode. Consequently, the TM-related intensity appears at a smaller fiber position along the detection coordinate, located closer to the excitation origin, which is consistent with the geometric arrangement illustrated in Figure 1.

### 3.3. Determination of Gain and Loss Parameters from Emission Data

To quantitatively assess the amplification behavior of the organic thin-film waveguide, the measured spatial emission profiles are evaluated with respect to the pump stripe geometry. The emission signal is divided into a pumped region and an unpumped region, corresponding to positions inside and outside the excited stripe. Within the pumped region, the guided modes exhibit an increase in intensity due to the material gain and spontaneous emission of the copolymer. In the unpumped region, the propagating modes decay exponentially as a result of waveguide losses. By analyzing the spatial evolution of the modal emission intensity in both regions, the modal gain and propagation loss can be extracted. The following section describes the procedure used for this decomposition and the subsequent parameter estimation method.

**Figure 5:**
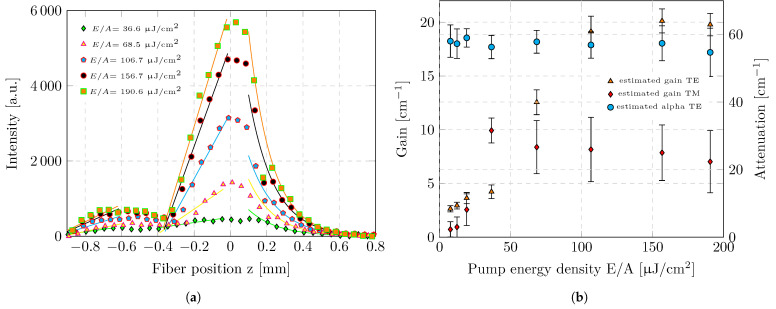
Growth and decay of the emission intensity along the pump stripe and corresponding fits of the signal. (**a**) Emission intensity at a selected wavelength (λ = 558 nm) as a function of the fiber position for various pump power densities. The plot includes linear fits describing the growth of the TM mode on the left side and the TE mode in the central region. The decay behavior of the TE mode on the right side of the plot is fitted using an exponential function. (**b**) Estimated gains and waveguide losses as a function of pump energy density at the selected wavelength (λ = 558 nm).

Data analysis was conducted individually for each emission wavelength. Figure 5a illustrates the measured emission intensity as a function of the fiber position for a selected wavelength (λ = 558 nm) at the centre of the expected ASE peak and various excitation densities. To distinguish between pumped and unpumped regions, the position of the maximum emission intensity was used as a reference. The transition from amplification to decay creates a local maximum. Data recorded at fiber positions smaller than the position of this maximum were assigned to the pumped region, whereas data at larger positions were attributed to the unpumped region. Data points in the transition region near the intensity maximum were excluded from the evaluation to avoid artifacts arising from the overlap of both regimes.

To determine the intrinsic waveguide losses, only the emission data assigned to the unpumped region were considered. In this regime, the guided mode propagates without optical amplification, and its intensity decreases due to waveguide losses. For each emission wavelength, the measured intensity was therefore fitted with a single-exponential decay function of the form

(7) $$I\left(\lambda ,z\right)=I_{0}\cdot e^{-\alpha \left(\lambda \right)\cdot z}$$

where *z* denotes the fiber position relative to the intensity maximum, $I_{0}$ is the intensity at the boundary between the pumped and unpumped regions, and α is the effective waveguide loss coefficient. Figure 5a shows the fitted decay functions for a representative emission wavelength of λ = 558 nm, plotted for different pump energy densities. The extracted loss coefficients (α(λ=558nm)) are summarized in Figure 5b. The loss coefficient remains constant, confirming that α is independent of the excitation density. This behavior was observed for all analyzed wavelengths. Reliable loss estimation could be achieved only for the TE mode. This behavior was observed for all analyzed wavelengths. For the TM mode, this fitting procedure was not feasible: in the unpumped region, its signal is overlapped by the rising TE-mode intensity, preventing an unambiguous extraction of an exponential decay.

In the pumped regime, the modal net gain can be estimated. According to Equation (4), the guided intensity follows a linear dependence of the form I=η·g·z. This linear growth arises from the combined contributions of spontaneous emission and optical amplification along the propagation direction. The diffracted light represents only a fraction of the internal guided intensity. Therefore, conversion to the absolute guided intensity requires knowledge of the diffraction efficiency of the outcoupling process. To determine the diffraction coefficients, we combined calculations of the discrete eigenvalues of the propagation constant with simulations performed using the open-source diffraction tool GD-Calc [28]. The simulations are based on a variant of the rigorous coupled-wave analysis (RCWA) method and explicitly account for coupling into the grating. They yield the modal power fractions (Π(σ/π,transmitted,m,λ)) distributed into the individual guided modes.

(8) $$g\left(\lambda ,\sigma /\pi \right)=\frac{1}{\eta_{modal}\left(\lambda \right)}\cdot \frac{1}{\Pi (\sigma /\pi ,transmitted,m,\lambda )}\cdot \frac{\Delta I_{meas}\left(\lambda \right)}{\Delta z}$$

Using the diffraction coefficients, modal amplification can then be described according to Equation (8). For improved stability, the directly measured diffracted intensity was replaced by the mean intensity gradient $\Delta I_{\mathrm{Meas}}/\Delta z$. This quantity was obtained by fitting a linear function to the measured intensity distribution using a least-squares approach. The separation of the modal gain requires normalization with the modal spontaneous emission $\eta_{modal}$. For this purpose, we used the isotropic spontaneous-emission background, which had been subtracted from the modal intensity beforehand. The isotropic background was extracted from the center of the excitation stripe to minimize edge-related spatial inhomogeneities. This approach assumes local invariance of the photoluminescence spectral shape within the central pumped region and within the investigated fluence range.

(9) $$\eta_{modal}\left(\lambda \right)=\frac{1}{2}\cdot \frac{4\cdot \pi \cdot A_{f}}{\Omega_{fiber}\cdot A_{pp}}\cdot \eta_{isotrop}$$

Equation (9) describes the spontaneous emission using a spatial correction and an unperturbed distribution into the π- and σ-polarized components. The first term of the equation results from the division of the spontaneous emission into these two polarization channels. The second term normalizes the spontaneous emission according to the solid angle captured by the fiber at the given measurement distance, accounting for the originally spherical emission pattern. Here, $A_{f}$ denotes the area of the waveguide core, while $A_{pp}$ represents the pumped area that lies within the acceptance cone of the fiber. For each pump energy density, $\eta_{\mathrm{modal}}$ was determined as the average value within the center of the excited stripe.

### 3.4. Extracted Parameters and Physical Interpretation

Having established the methodology for extracting the modal gain, loss, and spontaneous-emission contributions, we now turn to the physical interpretation of the obtained parameters. In the following section, we analyze how the gain and modal emission evolve with pump fluence, discuss the influence of diffraction and outcoupling, and relate the extracted quantities to the underlying photophysical processes in the organic waveguide.

**Figure 6:**
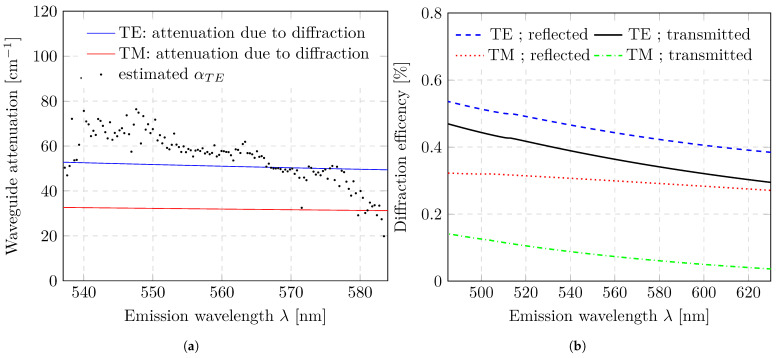
Modal waveguide attenuation. (**a**) Comparison between the estimated waveguide attenuation and the diffraction-induced waveguide attenuation. (**b**) Diffraction efficiency of the modal waveguide modes, calculated using the discrete waveguide eigenmodes and an RCWA approach implemented in the commercial software GD-Calc (Version 07/13/2019).

The first quantity we evaluate is the waveguide attenuation α (i.e., the propagation losses). Due to the integrated grating, the waveguide losses in our structure are relatively high. These losses originate from absorption, scattering, and diffraction-induced outcoupling. Since organic copolymers exhibit very low absorption in the emission wavelength range, the dominant contribution to α arises from grating-related losses rather than bulk absorption.To quantify this contribution, diffraction-efficiency simulations were performed. Using rigorous coupled-wave analysis (RCWA), diffraction efficiencies for reflected and transmitted modes were calculated as a function of wavelength for both TE and TM polarizations. The resulting efficiencies are shown in Figure 6a. Assuming negligible absorption and neglecting additional scattering losses, an effective waveguide attenuation model was derived from the simulated diffraction efficiencies. Figure 6b compares the calculated attenuation with experimentally extracted values for the TE mode, which were obtained from the exponential decay of the modal intensity in the unpumped region. Both the spectral trend and the absolute magnitude show good agreement between the simulation and the experiment. Compared to similar sample architectures reported in the literature, our extracted propagation losses (α) are approximately 50–90 times larger than typical reference values [29,30]. The estimated values of α obtained from our measurements exhibit a relatively large spread. During the evaluation step, we observed that the choice of the interval boundaries used for the exponential fit has a significant influence on the resulting attenuation. In particular, when data points close to the intensity maximum were included, a pronounced wavelength dependence on the extracted attenuation appeared. This effect was predominantly observed in spectral regions where the modal intensity was high. Furthermore, the wavelength-dependent variations in α then showed a dependence on the pump energy density. This suggests that, near the edges of the pumped stripe, the exponential decay of the guided mode may be perturbed by spatial inhomogeneities of the excitation. A possible explanation is that diffraction of the pump beam at the grating causes a fraction of the pump light to be redistributed into regions that should be unpumped, thereby affecting the measurement. As a consequence, a sufficiently large distance from the pumped region must be maintained when determining the attenuation. This requirement reduces the robustness of the loss determination step, particularly at low pump energy densities where the signal-to-noise ratio is limited. At higher fluences, the lower signal-to-noise ratio mitigates this effect, making the extraction of α considerably more reliable.

In the spectral emission range between approximately 540 nm and 580 nm, the attenuation decreases with increasing wavelength. Within this range, attenuation variations due to material-specific properties could not be observed. Outside this region, however, a pronounced decrease in α is observed, converging towards zero. This apparent reduction can likely be attributed to the limited detection window of the optical fiber: due to the decreasing detected emission intensity towards the spectral edges, the signal-to-noise ratio becomes insufficient to reliably resolve modal decay. While α exhibits some sensitivity to the pump energy density near the edges of the pumped stripe, this does not affect the gain calculation, as the normalization and modal amplification steps rely on the simulated diffraction coefficients.

**Figure 7:**
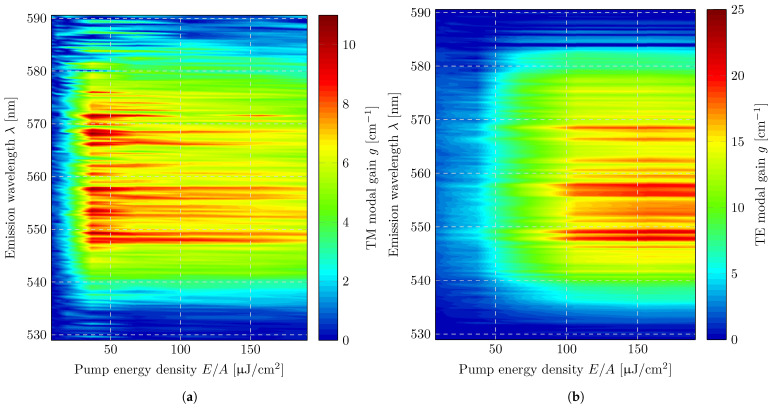
Two-dimensional representation (heatmap) of the net optical gain as a function of emission wavelength and excitation density. The data illustrate the spectral dependence on amplification and the onset of gains with increasing excitation: (**a**) π-polarized modal gain; (**b**) σ-polarized modal gain.

The modal gain was determined for both the TE and TM modes using Equations (8) and (9), following the procedure described in the previous section. The diffraction efficiencies required for gain extraction were taken from the waveguide simulations and incorporated into the evaluation. Figure 7a,b show the estimated gain of the TM mode (π-polarized emission) and the TE mode (σ-polarized emission), respectively, plotted as heatmaps as a function of pump energy density and emission wavelength. At low pump fluences, the gain values exhibit similar behavior for both polarizations, increasing steadily with pump energy density. However, at a relatively low fluence of approximately 40 μJ/cm^2^, the gain of the TM mode begins to saturate and remains essentially constant throughout the full measurement range up to 190 μJ/cm^2^. In contrast, the gain of the TE mode continues to increase nearly linearly with pump energy density and reaches saturation only at significantly higher fluences, around 120 μJ/cm^2^.

While the gain extraction method is intrinsically robust due to normalization to the simultaneously measured isotropic spontaneous emission, it is nevertheless instructive to assess the sensitivity of the extracted gain with respect to structural and material parameters that enter the diffraction efficiency range. Sensitivity was therefore evaluated using the chain rule, considering the TE-polarized mode at an emission wavelength of 550 nm as an illustrative example, since this mode exhibits a stronger dependence on structural variations than the TM mode. Within the investigated parameter range, the diffraction efficiency Π, obtained from numerical simulations, exhibits an approximately linear dependence on the grating’s height. The resulting relative sensitivity of the gain can be expressed as

(10) $$S_{g,d}^{rel}=\frac{1}{g}\cdot \frac{\partial g}{\partial d}=-\frac{1}{\Pi (d)}\cdot \frac{\partial \Pi }{\partial d}$$

For the considered grating heights, this results in a relative sensitivity on the order of −7% per nanometer, indicating a moderate but systematic decrease in the extracted gain with increasing diffraction efficiency. A similar analysis was performed for variations in the refractive index of the copolymer film. A relative deviation in the refractive index leads to a relative gain in sensitivity of approximately −4% per percent deviation, underscoring that material parameters can also noticeably affect the gain through their influence on diffraction efficiency. Film thickness itself only affects the gain indirectly via its impact on diffraction efficiency. However, deviations in refractive index and film thickness were accounted for by a model-based adjustment using an effective refractive index, which was estimated from the outcoupling direction of the diffracted modes as a function of wavelength. This procedure is described in detail in Ref. [21]. Across all wavelengths and excitation densities, the TM mode exhibits substantially lower gain values than the TE mode. The spectral dependence of the gain is similar for both polarizations, with full widths at half maximum of $FWHM_{TE}$ = 38.7 nm and $FWHM_{TM}$ = 36 nm and nearly symmetric profiles centered at $\lambda_{TE,center}$ = 559.7 nm and $\lambda_{TM,center}$ = 558.7 nm. Both modes display distinct local maxima at identical wavelengths, independent of polarization. Particularly prominent are three characteristic double-peak structures (at 548/549 nm, 556/558 nm, and 566/568 nm; see Figure 7b). These double peaks also correspond to the global gain maxima, reaching approximately 20 cm^−1^ for the TE mode and about 8 cm^−1^ for the TM mode.

The gain values extracted from the emission data fall within the typical range reported for organic copolymers and are consistent in magnitude with results obtained from conventional gain measurements. Furthermore, a comparison with the previously determined waveguide losses shows that the estimated gain remains well below the attenuation—an essential requirement for the validity of the applied method. Gain estimation relies on a linear fit of the decoupled emission as a function of the fiber position, which proves to be highly robust across the relevant measurement range. The boundaries of the effectively pumped region can be intuitively identified from the spatial emission profiles, ensuring a consistent and reproducible determination of the gain. On this basis, the following section examines how the extracted gain parameters relate to the resulting net amplification and discusses their implications for the overall outcoupling behavior of the system.

## 4. Discussion

The waveguide attenuation extracted from the experimental data shows values that are consistent with the expectations derived from the diffraction efficiency simulations. Both the absolute magnitude and the wavelength dependence of the estimated loss coefficient agree well with the simulated trends, indicating that the extracted values are physically plausible and primarily governed by the integrated grating. It is therefore important to emphasize that the experimentally obtained attenuation does not permit conclusions about the intrinsic material losses of the copolymer film. Organic copolymers typically exhibit only minor absorption within their emission band [31], whereas the grating-induced outcoupling strongly dominates the modal attenuation in our device geometry. Importantly, the dominance of grating-induced outcoupling represents a deliberate design choice rather than a limitation of the DEP method. By adjusting the grating strength and thus the effective waveguide attenuation, the method can be tailored to material systems with both low and high optical gains. This tunability of the modal losses enables broad applicability of the DEP approach across different gain regimes. Although the extraction of α is sufficiently robust for the present analysis, the measurement suffers from instability at low pump energy densities, particularly near the boundary of the pumped region. This effect is most likely related to diffraction of the pump beam at the razor-blade edges used to generate the excitation stripe, which can locally perturb the exponential decay of the guided mode. A top-hat beam profile—free from such diffraction artifacts—would therefore be beneficial for future studies, both to increase the stability of α extraction and to suppress systematic distortions at the stripe edges.

It should be noted that the present analysis does not explicitly account for diffraction of residual pump light at the grating. In the applied geometry, the pump beam traverses the strongly absorbing copolymer film before reaching the grating such that the transmitted pump intensity is strongly reduced. Consequently, any feedback or redistribution of pump light into nominally unpumped regions is expected to be negligible for the film thicknesses investigated here. However, for very thin or weakly absorbing films, pump-light diffraction at the grating could, in principle, contribute to an apparent increase in the detected emission signal and thus influence the gain evaluation. A quantitative assessment of this effect is beyond the scope of the present work but should be considered in future studies. The modal gain evaluation is based on the normalization of the guided emission to the isotropic spontaneous emission. This normalization approach is a central advantage of the DEP method: by dividing modal intensities by the simultaneously measured isotropic emission, all absolute-intensity-related factors—including waveguide coupling efficiencies, the unknown responsivity of the spectrometer, timing—cancel out. As a result, only geometric parameters must be considered explicitly, in particular those related to the spatial emission distributions of guided versus isotropic components. This renders the method comparatively insensitive to experimental uncertainties and allows direct and robust extraction of the modal gain.

The obtained gain values lie within the expected range for organic copolymers, consistent with results obtained using ASE-based measurement techniques, the variable-stripe-length (VSL) method, and classical stripe-length experiments. Earlier studies [29,32] have reported similar gain coefficients in F8BT. Furthermore, the spectral shape of the extracted gain—its FWHM abd central wavelength— agrees well with previous ASE studies on F8BT [25,26,32]. Both the TE and TM modes exhibit nearly identical spectral envelopes and peak positions, indicating that the underlying emissive processes are polarization-independent. Nevertheless, substantial differences in the absolute gain values between the TE and TM modes are observed, with the TM-mode gain being significantly smaller. It is well known that TM modes tend to exhibit stronger overlap with cladding layers, which inherently reduces their effective modal gain relative to TE modes in waveguide structures. Thin films of F8BT and related copolymers are also known to exhibit molecular alignment effects [29,33,34] in a thickness range <200 nm. Such anisotropy not only influences polarization-dependent optical constants such as the refractive index but also affects the gain coefficient and modal losses, indicating that chain orientation may directly contribute to the polarization-dependent gain observed in our experiment.

Gain saturation effects have been reported in the literature for excitation fluences comparable to those used here, but they are typically associated with longer excitation stripe lengths of several millimeters. In the present work, the stripe length is limited to 0.5 mm, which is considerably shorter than the characteristic propagation lengths at which saturation due to stimulated emission and excited-state depletion is usually discussed. Saturation may also occur due to limited or nonlinear absorption of the pump radiation at higher fluences. Such effects could, in principle, influence the applicability of a linear fitting approach. However, saturation effects were not explicitly investigated in this study, and a dedicated analysis would be required to assess their quantitative impact. The spectral shape of the extracted gain reveals a distinct fine structure that resembles a web of narrowband features with varying prominence. Rather than forming a smooth broadband amplification band, the gain spectrum appears as the superposition of multiple narrow contributions. This behavior suggests that the observed features may correspond to different energetically separated transitions, each contributing with slightly different strengths to the overall amplification profile. The measurement geometry imposes an additional constraint on the observable spectral range: because the diffraction angle of the outcoupled light depends on the emission wavelength, only a limited number of diffracted modes can be detected at a fixed measurement angle. The accessible bandwidth is determined by the fiber’s acceptance angle and by the wavelength-dependent modal propagation constant. For the present setup, this results in a measurable range of approximately 70 nm. The gain was evaluated within the 530–590 nm window, which lies close to the upper limit of the accessible bandwidth. Consequently, gain values recorded at the spectral boundaries may be affected by reduced coupling efficiency. Future studies could overcome this limitation by combining measurements at several detection angles, thereby allowing the full gain profile—including the outer regions of the ASE band—to be resolved more accurately. The present analysis assumes that only the fundamental guided mode contributes to the diffracted emission signal. Due to the chosen film thickness and refractive index contrast, higher-order guided modes as well as higher diffraction orders are not supported.

## 5. Conclusions

In this work, the diffracted emission profile (DEP) method was introduced and applied to determine the optical gain of an organic copolymer thin film. The method enables the spatially resolved characterization of amplification processes by monitoring the diffracted portion of guided emission from an integrated one-dimensional grating.

Although the presented measurement approach requires a comparatively sophisticated experimental configuration, it offers a unique and conceptually powerful route toward quantifying modal gain in organic thin-film waveguides. Optical alignment is critical: both the orientation of the pump stripe relative to the grating and the positioning of the detection fiber relative to the diffracted modes must be realized with high accuracy, and the extraction of absolute gain values further relies on simulated diffraction efficiencies.

However, despite these practical challenges, the methodology provides several decisive benefits that are not accessible with established gain-measurement techniques. Most notably, it enables the quantitative estimation of the modal gain even in regimes where the waveguide losses exceed the amplification. This capability distinguishes the method from classical variable-stripe-length measurements which require a positive net gain. In contrast, the present method decouples the modal emission from the isotropic spontaneous emission, allowing absolute modal gain to be inferred even when no net amplification occurs and the system remains loss-dominated. This feature broadens the accessible parameter space for gain studies in organic semiconductors. It allows meaningful gain characterization in materials, layer stacks, or device geometries that would otherwise remain experimentally inaccessible.

Moreover, the sample architecture closely mirrors that of a practical DFB laser, making the extracted modal gain directly relevant for device design. The technique also naturally provides polarization-resolved gain information without additional components, since TE and TM modes are separated through their diffraction angle outcoupling. Together, these advantages establish the method as an informative tool for studying modal gain in organic thin-film systems, particularly in situations where conventional approaches fail and where early, accurate insight into the interplay of gain and loss is essential.

## Data Availability

The original contributions presented in this study are included in the article. Further inquiries can be directed to the corresponding author.
